# Decreased Hemoglobin Concentration and Iron Metabolism Disorder in Periodontitis: Systematic Review and Meta-Analysis

**DOI:** 10.3389/fphys.2019.01620

**Published:** 2020-01-31

**Authors:** Donglei Wu, Zhengshen Lin, Shiwei Zhang, Fengdi Cao, Defeng Liang, Xincai Zhou

**Affiliations:** ^1^Department of Stomatology, Jinan University-Affiliated Shenzhen Baoan Women's and Children's Hospital, Shenzhen, China; ^2^Department of Stomatology, Shunde Hospital, Southern Medical University (The First People's Hospital of Shunde Foshan), Foshan, China; ^3^Department of Stomatology, The First Affiliated Hospital Jinan University, Guangzhou, China

**Keywords:** periodontal diseases, periodontitis, chronic inflammation diseases, anemia of inflammation, hemoglobin parameters iron metabolism, hepcidin, meta-analysis

## Abstract

**Background:** Periodontitis is a chronic inflammatory disease with a possible infectious component. Anemia of inflammation (AI) occurring in various chronic diseases alters the hemoglobin (Hb) concentration and iron status. Currently, the association between periodontitis and AI is still controversial. The aim of this study was to assess the alterations of the level of hematological parameters and iron metabolism markers in patients with or without periodontitis.

**Methods:** Electronic databases (MEDLINE, EMBASE, and Cochrane) were searched to identify publications about anemia and periodontitis. Subgroup analyses regarding gender, extent of periodontitis, and sample size were performed using STATA 12.1.

**Results:** Sixteen studies were included in this meta-analysis. Pooled results showed a decrease in Hb [standardized mean difference (SMD) = −0.76, 95% CI = (−1.15, −0.37)], red blood cell [SMD = −0.69, 95% CI = (−1.09, −0.29)], hematocrit [SMD = −1.13, 95% CI = (−1.69, −0.57)], mean corpuscular volume [SMD = −0.16, 95% CI = (−0.32, −0.01)], and mean corpuscular Hb [SMD = −0.16, 95% CI = (−0.28, −0.04)], but upregulation in erythrocyte sedimentation rate [SMD = 0.63, 95% CI = (0.06, 1.19)]. In addition, patients with periodontitis had a higher level of hepcidin [SMD = 0.59, CI = (0.05, 1.12)] and decreased level of transferrin [SMD = −4.6, CI = (−13.1, −3.90)], with high heterogeneity.

**Conclusion:** This meta-analysis indicates that periodontitis decreases Hb concentration and disturbs the balance of iron metabolism, which confirms strength of association between periodontitis and the development tendency of AI, especially for severe periodontitis. More unbiased cohort studies with larger sample sizes are still warranted to make a definitive judgment in the future.

## Introduction

Anemia, as a global major health burden, affects 1.62 billion people, especially in the low- and middle-income countries (McLean et al., 2009). Despite many efforts made to tackle this burden, it retains a high prevalence around the world. Generally, anemia of inflammation (AI), a hallmark of chronic and persistent inflammatory diseases with prolonged immune activation (Weiss et al., 2019), is characterized by the insufficient production of erythropoietin (EPO) and decreased response of erythroid progenitors to EPO (Spoto et al., 2019). In addition, AI inhibits the release of iron from the body stores and alters the iron status after being stimulated by hepcidin (Schumann and Solomons, 2017). A number of diseases might ultimately lead to AI, including chronic kidney disease, chronic pulmonary disease, and congestive heart failure (Weiss et al., 2019).

Periodontitis, as a common chronic infectious disease in dentistry, is mediated by the immune inflammatory response to the accumulated periodontal pathogens in the periodontal tissues, which involves both innate and acquired immunity (Cekici et al., 2014). It decreases the supporting bone level and eventually leads to tooth loss. It is widely known that periodontal diseases (PDs) not only induce local inflammation but also lead to higher systematic inflammation such as vascular dysfunction and cardiovascular events (Nibal et al., 2019). Recent evidence demonstrated that PD (chronical periodontitis and aggressive periodontitis) tended to lead to anemia. A study by Anand et al. found that patients with generalized aggressive periodontitis, in comparison with health controls, were susceptible to lower red blood cell (RBC) counts and lower hemoglobin (Hb) levels (Anand et al., 2014). However, there were several references in argument of a non-coincidental association (Enhos et al., 2009; Carvalho et al., 2016). Hence, the aim of the present study was to evaluate the alterations of the level of hematological parameters and iron metabolism markers in patients with or without periodontitis.

## Materials and Methods

This study was conducted following the Preferred Reporting Items for Systematic Reviews and Meta-analysis guidelines and the outlines of PICOS: adults (P = patients), periodontitis (I = intervention/exposure), patients without periodontitis (C = comparison), alterations of hematological parameters and iron metabolism markers (O = outcome), and observational studies (S = study design). We mainly concentrated on solving the following clinical question: Do patients with periodontitis have a decreased level of Hb concentration and serum iron metabolism disorder?

### Search Strategy

Three databases including MEDLINE, EMBASE, and Cochrane Central were searched from inception to April 2019 for studies published in English. The search terms were as follows: (periodontitis OR “periodontal diseases” OR “chronic periodontitis”) AND (anemia OR “hematological parameters” OR hemoglobin OR Hb OR “red blood cell counts” OR RBC OR HCT OR hematocrit OR MCV OR “mean corpuscular volume” OR MCH OR “mean corpuscular hemoglobin” OR MCHC OR “mean corpuscular hemoglobin concentration” OR ESR OR “erythrocyte sedimentation rate” OR iron OR transferrin OR ferritin OR hepcidin). All data extraction was conducted by two investigators independently (SW and FD).

### Eligibility and Exclusion Criteria

The eligibility criteria were as follows: (1) study design: observational studies (cross-sectional); (2) studies that evaluate the association between alterations of the level of hematological parameters and iron metabolism markers and periodontitis; (3) studies that include a diagnosis of periodontitis with clinical or radiological examination; (4) periodontitis as an exposure in related research; and (5) studies where the values of mean ± standard deviation in the evaluated parameters were provided.

Furthermore, the exclusion criteria in this meta-analysis were as follows: (1) subjects with inherited anemias such as Fanconi anemia, sickle cell anemia, and β-thalassemia disease; (2) aggressive periodontitis; (3) absence of the definition of periodontitis; (4) subjects with other chronic inflammatory diseases or microbial infections (virus, fungus, and bacteria); (5) subjects with systematic diseases such as diabetes, kidney diseases, and cancer; (6) absence of control groups; (7) no blood samples used, such as those studies using only gingival crevicular fluid (GCF); (8) case reports, reviews, commentaries, and preclinical studies (animal and *in vitro* studies); and (9) pregnant or lactating women.

### Data Extraction and Collection

Information regarding the included publications were extracted, including the last name of the first author, year of publication, country of study, sample size, characteristics of the included subjects, definition of periodontitis, outcome measures, and positive outcome measures.

### Quality Assessment

Quality assessment was conducted using the Newcastle–Ottawa scale (NOS), which is judged based on three broad perspectives (selection, comparability, and exposure/outcome), with a maximum of nine scores. This was assessed by two authors (DL and XC) independently. Discrepancies in the NOS scores were resolved by the third author (ZS). The NOS scores of the studies included in this meta-analysis are presented in **Table 3**.

### Statistical Analysis

Meta-analysis was conducted to evaluate the association between periodontitis and the occurrence of anemia. With regard to the continuous variable presented in this meta-analysis, the SMD and 95% confidence interval (CI) were computed using the random effects model. The SMD and 95% CI represent the pooled results of this study. To quantify the heterogeneity in this meta-analysis, the heterogeneity chi-square test and *I*^2^ index were calculated. *I*^2^ > 75% is generally considered as high heterogeneity. Subgroup analysis with the preplanned confounders (gender, sample size, and severity of periodontitis), among the included observational studies, was performed to further explore the origin of heterogeneity. Sensitivity analyses were carried out to assess the individual influences of each included study. Publication bias was assessed via funnel plot and Egger regression intercept. All the statistical analyses were performed using STATA version 12.1.

## Results

### Study Selection for Meta-Analysis

**Figure 1:**
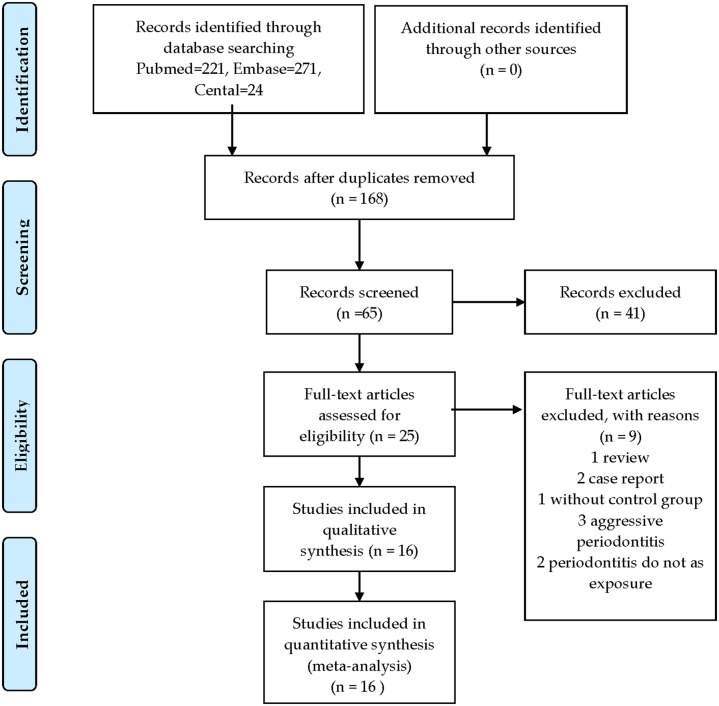
Flow diagram of study selection.

A flow diagram of the study selection is presented in Figure 1. A total of 516 articles were identified and screened, and 168 were selected. Nine studies were excluded after screening the full text, including three on aggressive periodontitis, two that did not involve exposure to periodontitis, one that lacked a control group, two case reports, and one review. Besides, no cohort or case–control studies could be retrieved. At the end, 16 cross-sectional studies (including blood parameters and iron metabolic markers) were included in this systematic review and meta-analysis.

### Characteristics of Included Studies

The characteristics of the included studies are summarized in Table 1. A total of 1,423 patients were included to assess the association between periodontitis and the risk of anemia. Approximately two-thirds of the studies were conducted in India, and only four included only male subjects. Papers by Prakash (Prakash et al., 2012; Rao et al., 2013) and Hutter et al. (2001) evaluated the association between anemia and gender. Twelve studies were involved in assessment of the effect of periodontitis on hematological parameters, including Hb, RBC counts, hematocrit (HCT), mean corpuscular volume (MCV), mean corpuscular Hb (MCH), mean corpuscular Hb concentration (MCHC), and erythrocyte sedimentation rate (ESR) (Hutter et al., 2001; Gokhale et al., 2010; Naik et al., 2010; Prakash et al., 2012; Chakraborty et al., 2014; Kolte et al., 2014; Patel et al., 2014; Khan et al., 2015; Latha et al., 2015; Anumolu et al., 2016; Carvalho et al., 2016; Nibal et al., 2019).

**Table 1A:** Characteristic of included studies.

**References**	**Country**	**Male/female**	**Demographic of subjects**	**Complete blood count**	**Iron metabolism marker**	**Positive results** **(*p* < 0.05)**
Hutter et al. (2001)	Netherlands	TG: 19/20 CG: 24/18	Severe CPD	HB, RBC, HCT, MCV, MCH, MCHC	NA	HB, RBC, HCT,
Naik et al. (2010)	India	All male	30–60 years ≥20 teeth BMI: 18.5–25	HB,RBC, HCT, MCV, MCHC, ESR	NA	HB,RBC, HCT, MCV, MCHC, ESR
Gokhale et al. (2010)	India	All male	25–50 years	HB, RBC, HCT MCV, MCH, MCHC	NA	RBC, HCT, HB
Prakash et al. (2012)	India	TG: 51/39 CG: 27/23	35–65 years	HB, HCT MCV, MCH, MCHC, ESR	sFe, sFerritin	NA
Patel et al. (2014)	India	All male	20–50 years	HB, RBC, HCT MCV, MCH, MCHC	NA	HB, RBC, HCT, MCV MCH, MCHC
Chakraborty et al. (2014)	India	TG: 9/11 CG: 12/10	TG: 33.13 ± 6.38 (24–42 years) CG: 36.18 ± 5.15 (24–42 years)	HB	sFerritin	HB, ferritin
Khan et al. (2015)	India	All male	≤ 55 years	HB, RBC, HCT, MCV, MCH, MCHC	NA	HB, RBC, HCT, MCV, MCH, MCHC
Latha et al. (2015)	India	TG: 7/7 CG: 5/9	40–50 years	HB, RBC, HCT MCV, MCH, MCHC, ESR,	sFerritin	NA
Anumolu et al. (2016)	India	TG: 33/17 CG: 27/23	20–55 years	HB, RBC, HCT MCV, MCH, MCHC, ESR	NA	HB, RBC,
Carvalho et al. (2016)	Brazil	NA	NA	HB, MCV, MCH, HCT	sFe, sFerritin Transferrin Hepcidin	NA
Kolte et al. (2014)	India	TG: 47/53 CG: 42/58	30–60 years	HB, MCV, MCH	NA	RBC MCHC
Nibal et al. (2019)	UK	TG: 50/71 CG: 104/121	TG: 45.12 ± 10.04 CG: 37.65 ± 11.52	HB, RBC, HCT MCV, MCH, MCHC	NA	NA
Shirmohamadi et al. (2016)	Iran	TG: 11/9 CG: 9/11	TG: 42.13 ± 8.36 (28–56 years) CG: 33.1 ± 4.41 (25–42 years)	NA	Transferrin	Transferrin
Thomas et al. (2013)	India	NA	35–60 years	NA	sFe	sFe
Craig et al. (2003)	India	NA	TG: 38.7 ± 1.3 CG: 29.9 ± 1.1	NA	sFe	
Guo et al. (2018)	China	TG: 9/11 CG: 14/8	CG: 52.45 ± 10.01 TG: 58.09 ± 9.97	NA	Hepcidin	Hepcidin

*CPD, chronic periodontal disease; CG, control group; EC, erythrocyte count; ESR, erythrocyte sedimentation rate; HCT, hematocrit; HB, hemoglobin; MCH, mean corpuscular hemoglobin; MCHC, mean corpuscular hemoglobin concentration; MCV, mean corpuscular volume; RBC, red blood cell count; RDW, red cell distribution width; TG, test group; NA, not available. Values are given as mean ± SD unless the variable is not normally distributed (indicated by the superscript letter, in which case (median, range) are also given*.

**Table 1B:** Characteristic of included studies.

**References**	**Case/control**	**Case/control ratio estimation**	**Case/control matching**	**Criteria of CPD**	**Criteria of Control**	**Temporal association**	**NOS**
Hutter et al. (2001)	39/42	NA	Yes	BL ≥ 50% (≥7 teeth)	CEJ to alveolar bone crest <2 mm	In parallel	6
Naik et al. (2010)	15/15	NA	Yes	CAL: 30% sites ≥ 5 mm, or most sites ≥ 6 mm	CAL: 0% sites	In parallel	7
Gokhale et al. (2010)	30/30	NA	NA	PD ≥ 6 mm (30% sites) BL > 50%	PD <3 mm	In parallel	5
Prakash et al. (2012)	90/50	NA	Yes	CAL ≥ 2 mm	PD <3 mm CAL: 0% sites; without BL	In parallel	6
Patel et al. (2014)	50/50	NA	Yes	PD ≥ 2 mm and AL ≥ 2 mm (30% sites)	PD <3 mm CAL: 0% sites	In parallel	6
Chakraborty et al. (2014)	20/22	A significant difference in BOP% with 80% power and two-sided 0.05 level of significance	NA	CAL ≥ 4 mm, PD ≥ 5 mm (≥2 interproximal sites)		In parallel	6
Khan et al. (2015)	20/20	NA	Yes	PD ≥ 4 mm and CAL ≥ 5 mm	Clinically healthy gingival	In parallel	6
Latha et al. (2015)	14/14	NA	NA	PD ≥ 4 mm and CAL ≥ 1 mm	PD <3 mm CAL: 0% sites	In parallel	6
Anumolu et al. (2016)	50/50	NA	NA	PD ≥ 5 mm (30% sites) and CAL ≥ 2 mm	GI: 0–1	In parallel	6
Carvalho et al. (2016)	33/30	NA	NA	PD ≥ 5 mm and AL ≥ 6 mm	Absence of periodontal infection	In parallel	6
Kolte et al. (2014)	100/100	NA	NA	PD ≥ 5 mm	PD ≤ 3 mm	In parallel	6
Nibal et al. (2019)	121/225	NA	NA	PD and CAL ≥ 5 mm	PD and CAL <5 mm (≥20 teeth)	In parallel	6
Shirmohamadi et al. (2016)	20/20	α = 0.05 and a statistical power of 80%	NA	CAL ≥ 4 mm, PD ≥ 5 mm (≥2 interproximal sites)	PD <3 mm CAL: 0% sites	In parallel	6
Thomas et al. (2013)	50/50	NA	NA	CAL ≥ 4 mm (30% sites)	PD ≤ 3 mm GI ≤ 2 (≥20 teeth)	In parallel	6
Craig et al. (2003)	44/25	NA	Yes	CAL > 3 mm, PD > 3 mm (≥4 sites)	CAL <3 mm (≥24 teeth)	In parallel	6
Guo et al. (2018)	22/22	NA	NA	BL ≥ 30%, CAL ≥ 5 mm, PD ≥ 5 mm	Clinically healthy gingival	In parallel	6

*BL, alveolar bone loss; CAL, clinical attachment level; CEJ, cemento-enamel junction; CPD, chronic periodontal disease; CG, control group; GI, gingival index; NA, not available; PD, probing depth*.

In addition, iron metabolism markers reflecting AI (serum iron, transferrin, and ferritin) and an iron state regulator (hepcidin) were included in this systematic review and meta-analysis. Four publications evaluated the effect of periodontitis on serum iron (Craig et al., 2003; Prakash et al., 2012; Thomas et al., 2013; Carvalho et al., 2016). Two articles on transferrin (Carvalho et al., 2016; Shirmohamadi et al., 2016), four references on ferritin (Prakash et al., 2012; Chakraborty et al., 2014; Latha et al., 2015; Carvalho et al., 2016), and two studies on hepcidin (Carvalho et al., 2016; Guo et al., 2018) were also included. Only one study assessing the effect of periodontitis on hepcidin in the GCF was excluded (Enhos et al., 2009).

### Meta-Analysis Results

#### Association Between Periodontitis and Alterations of Hematological Parameters

Our results showed that periodontitis increased the tendency to develop AI. Pooled analysis of 12 studies is presented in Table 2. Hematological parameters (Hb, RBC counts, HCT, MCV, MCH, MCHC, and ESR) were involved in this systematic review and meta-analysis. Periodontitis seemed to decrease the level of Hb [SMD = −0.76, 95% CI = (−1.15, −0.37)], RBC [SMD = −0.69, 95% CI = (−1.09, −0.29)], HCT [SMD = −1.13, 95% CI = (−1.69, −0.57)], MCV [SMD = −0.16, 95% CI = (−0.32, −0.01)], and MCH [SMD = −0.16, 95% CI = (−0.28, −0.04)]. Furthermore, ESR [SMD = 0.63, 95% CI = (0.06, 1.19)] was found to be upregulated in patients with periodontitis. Nevertheless, significant heterogeneity was observed in all the hematological parameters.

**Table 2:** Meta-analysis of the association between periodontitis and the risk of anemia.

**Classification**	**Number** **of subjects**	**SMD (95% CI)**	***I*^**2**^ (%)**	***P*-value**
**HEMATOLOGICAL PARAMETERS**				
Hb	12	**−0.76 (−1.15**, **−0.37)**	89.9	<0.01
RBC	11	**0.69 (−1.09,−0.29)**	89.8	<0.01
HCT	10	**−1.13 (−1.69**, **−0.57)**	93.4	<0.01
MCV	11	**−0.16 (−0.32**, **−0.01)**	34.0	0.03
MCH	9	**−0.16 (−0.28,−0.04)**	0.00	<0.01
MCHC	10	**–**0.04 (**–**0.55, 0.47)	93.6	0.88
ESR	4	**0.63 (0.06, 1.19)**	79.9	0.03
**IRON BIOMARKERS**				
Serum iron	4	**–**1.06 (**–**2.27, 0.15)	96.3	0.09
Ferritin	4	0.75 (**–**0.39, 1.90)	94.2	0.19
Transferrin	2	**−4.6 (−13.1**, **−3.90)**	98.4	0.02
Hepcidin	2	**0.59 (0.05, 1.12)**	45.6	0.03

*Bold means low heterogeneity in significance tests*.

*BL, radiographic bone loss; CAL, clinical attachment loss; CI, confidence interval*.

**Table 3:** Quality assessment of the included study in this meta-analysis.

**References**	**Study design**	**Selection**	**Comparability**	**Exposure/outcome**	**Total of 9**
Carvalho et al. (2016)	Cross-section	2	1	3	6
Chakraborty et al. (2014)	Cross-section	2	1	3	6
Gokhale et al. (2010)	Cross-section	1	1	3	5
Kolte et al. (2014)	Cross-section	2	1	3	6
Naik et al. (2010)	Cross-section	3	1	3	7
Prakash et al. (2012)	Cross-section	2	1	3	6
Patel et al. (2014)	Cross-section	2	1	3	6
Anumolu et al. (2016)	Cross-section	2	1	3	6
Latha et al. (2015)	Cross-section	2	1	3	6
Hutter et al. (2001)	Cross-section	2	1	3	6
Khan et al. (2015)	Cross-section	2	1	3	6
Nibal et al. (2019)	Cross-section	2	1	3	6

#### Association Between Periodontitis and Alterations of Iron Metabolism Markers

A combined analysis including the association between periodontitis and the metabolic markers of iron showed that periodontitis significantly decreased the transferrin level [SMD = −4.6, 95% CI = (−13.1, −3.90)]. Of great interest was that patients with periodontitis had a higher level of hepcidin [SMD = 0.59, 95% CI = (0.05, 1.12)]. However, there was significant heterogeneity in this pooled result (Table 2). Sensitivity analysis and publication bias could not be performed due to the limited number of included studies. No significant relationship was noted among the remaining parameters.

### Subgroup Analysis

**Figure 2:**
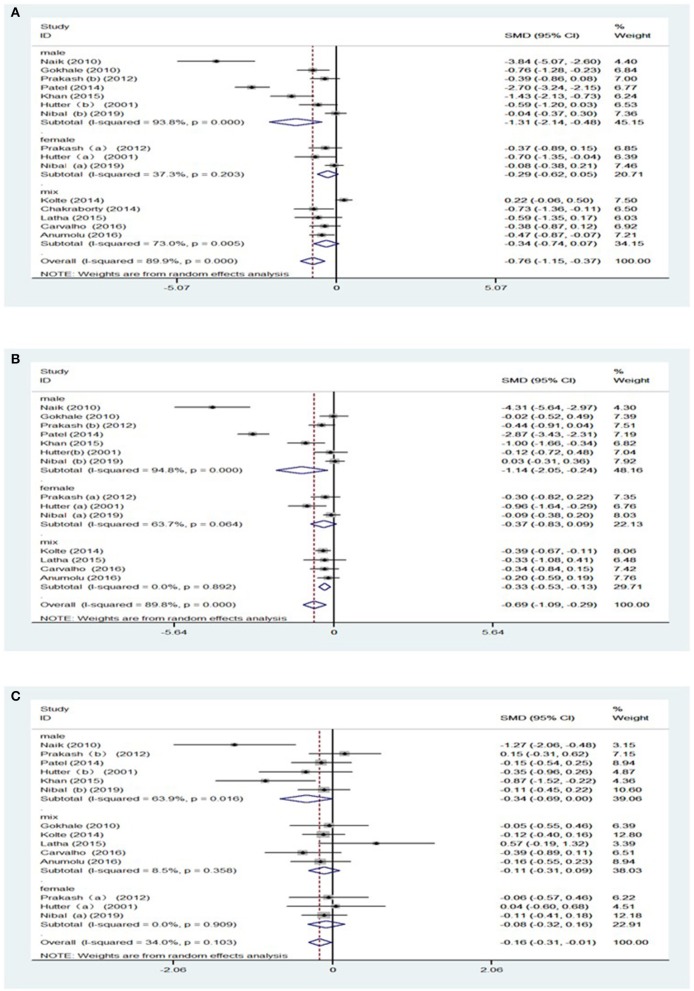
Forest plot of the studies regarding gender. **(A)** Forest plot of the studies regarding association between Hb and gender; **(B)** Forest plot of the studies regarding association between RBC counts and gender.; **(C)** Forest plot of the studies regarding association between MCV and gender. SMD, standardized mean difference; CI, confidence intervals; (a) means of female participants in the studies, (b) means of male participants in studies.

Subgroup analyses were conducted to further explore the sources of high heterogeneity. Owing to the discrepancy in the reference ranges of blood parameters with gender, we conducted a subgroup analysis for gender, as shown in Table 2, Supplementary Tables 1, 2. Hb level [SMD = −0.29, 95% CI = (−0.63, 0.05), *I*^2^ = 36.5%, Figure 2A] and RBC [SMD = −0.37, 95% CI = (−0.83, 0.09), *I*^2^ = 63.7%, Figure 2B] showed no significant differences on comparing women with periodontitis with those with good periodontal health. However, men with periodontitis showed decreased MCV [SMD = −0.13, 95% CI = (−0.69, 0.00), *I*^2^ = 63.9%, Figure 2C], when compared to men without periodontitis (Supplementary Table 3). In addition, subgroup analysis of the gender failed to figure out the origin of heterogeneity in MCH and HCT (Supplementary Figures 1, 2).

**Figure 3:**
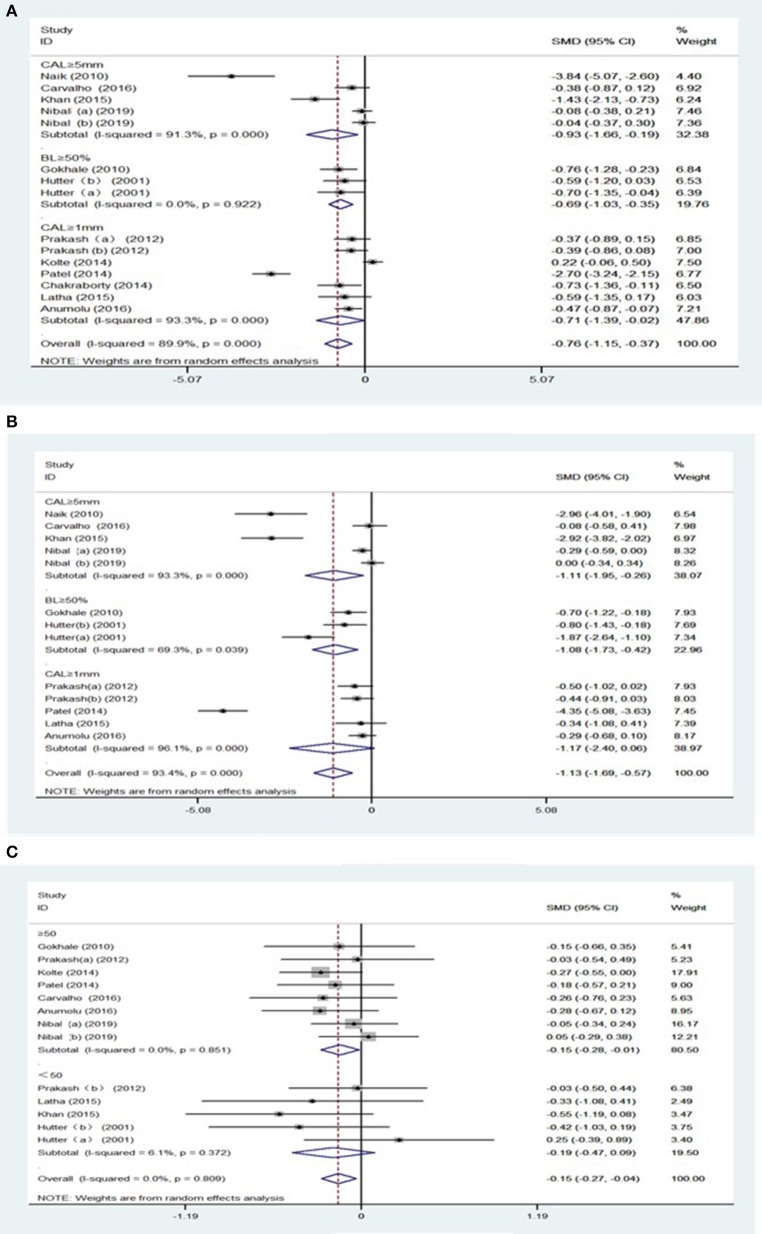
Forest plot of the studies regarding the extent of periodontitis and sample sizes. **(A)** Forest plot of the studies regarding association between the extent of periodontitis and Hb; **(B)** Forest plot of the studies regarding association between the extent of periodontitis and HCT; **(C)** Forest plot of the studies regarding association between sample sizes and MCH.

Furthermore, secondary analyses were conducted for the severity of periodontitis. Significantly, the pooled result of Hb level [SMD = −0.69, 95% CI = (−1.03, −0.35), *I*^2^ = 0.00%, Figure 3A] were decreased in subjects with over 50% of radiographic bone loss (BL), without heterogeneity between the studies. Likewise, patients with BL ≥ 50% showed lower HCT levels with moderate heterogeneity between the studies [SMD = −1.08, 95% CI = (−2.64, −0.42), *I*^2^ = 69.3%, Figure 3B] (Supplementary Table 4).

Subgroup analyses were subsequently carried out due to the great difference in the sample size among studies. There was a decrease in MCH [SMD = −0.15, 95% CI = (−0.28, −0.04), *I*^2^ = 0.00%, Figure 3C] in patients with periodontitis, as shown in studies with sample size larger than 50 (Supplementary Table 5).

### Sensitivity Analysis

Sensitivity analysis was carried out by excluding the included studies gradually to identify the individual influence in pooled results. Pooled analysis on Hb was affected by the studies conducted by Kolte et al. (2014) and Patel et al. (2014) to a large extent (Supplementary Figure 3a). Otherwise, pooled results of the RBC count and HCT changed after omitting the study by Patel (Supplementary Figures 3b,c). In case of iron biomarkers, the study conducted by Chakraborty et al. (2014) affected the pooled results of serum ferritin, while the study conducted by Craig et al. (2003) altered the pooled level of serum iron.

### Publication Bias

Publication bias in this meta-analysis was noted in the study evaluating the association between periodontitis and Hb (Supplementary Figure 4) and HCT (Supplementary Figure 5). No evidence of significant publication bias was found with regard to RBC (Supplementary Figure 6), MCV (Supplementary Figure 7), MCH (Supplementary Figure 8), and ESR (Supplementary Figure 9).

## Discussion

### Periodontitis Tends to Decrease the Hemoglobin Concentration

This systematic review and meta-analysis showed that patients with periodontitis demonstrated a lower level of Hb, RBC, and MCV with an increased ESR level. Although heterogeneity remained, this pooled result confirmed the association between periodontitis and the tendency of AI.

The outcome of this systematic evidence revealed that the extent of periodontitis affects the alteration of blood parameters, especially the degree of bone loss. This suggests that patients with severe periodontitis have a higher possibility of developing AI. This result is in agreement with a study conducted by Hutter et al. (2001) and Khan et al. (2015). In addition, aggressive periodontitis has a higher tendency to cause the patient to develop AI when compared to chronic periodontitis (Anand et al., 2014). Improvement of hematological indices were observed after periodontal treatment (Chakraborty et al., 2014).

Sensitivity analysis suggests that individual studies by Naik et al. (2010) had a strong impact on the stable of random-effect model in this meta-analysis. The possible explanation is that inclusion in this study was limited to patients with general severe and chronic periodontitis, which is relatively more severe than that in the other included studies.

### Periodontitis Alters Iron Status

To evaluate the effect of periodontitis on the iron status, circulating iron (serum transferrin and iron) and iron reserves (ferritin) were being investigated. Increased hepcidin and ferritin and decreased transferrin highly implicates the occurrence of AI, which is characterized by a dysfunction in iron metabolism including hypoferremia, hyperferritinemia, and increased hepcidin.

#### Periodontitis Increases the Level of Hepcidin

Hepcidin is the master regulator of iron homeostasis induced by numerous cytokines. It promotes iron retention in macrophages by degrading ferroportin1 and inhibits dietary iron absorption in the duodenum (Weiss et al., 2019). Patients with periodontitis demonstrated a higher level of hepcidin in serum and saliva than healthy controls. After periodontal treatment, the level of hepcidin decreased (Carvalho et al., 2016; Guo et al., 2018). In addition, periodontitis with chronic kidney disease or type 2 diabetes mellitus showed a higher level of hepcidin in comparison with isolated cases of periodontitis (Vilela et al., 2011; Guo et al., 2018).

However, more robust evidence is further warranted due to its high heterogeneity in pooled results. It was reported that the production of prohepcidin, the prohormone of hepcidin, was induced by chronic periodontal disease, and its level decreased after 3 months of periodontal therapy (Vilela et al., 2011). This may offer an explanation for the alteration of iron homeostasis.

#### Periodontal Pathogens Assimilate Iron From Transferrin

Serum transferrin, as a negative acute phase protein, reduces infection during inflammation (Jain et al., 2011). Furthermore, serum transferrin enables firm binding to Fe^3+^ and carries it to the target cell, creating a low Fe^3+^ concentration environment to inhibit the proliferation of microorganisms. Interestingly, there are numerous periodontal pathogens, which assimilate iron from transferrin via a siderophore-independent system (Duchesne et al., 1999). *Prevotella nigrescens, Prevotella intermedia*, and *Campylobacter rectus* (Grenier and Tanabe, 2011) were found to have the ability to obtain iron with their transferrin-binding activity (Duchesne et al., 1999; Gokhale et al., 2010). Furthermore, Goulet revealed that Arg-gingipains A and B, the virulence factors secreted by *Porphyromonas gingivalis*, enabled degradation of human transferrin and resulted in the production of free iron, which supplied various forms of iron and peptides for bacterial cells. In addition, the release of iron could result in the destruction of tissues by catalyzing the formation of toxic HO·(Goulet et al., 2004). Shirmohamadi found that the transferrin levels were decreased in patients with increased probing depth and returned to normal levels when the patients were subjected to periodontal treatment for 3 months (Shirmohamadi et al., 2016). This finding is consistent with that of Olśanska-Seidlová et al. (1989). Low serum transferrin is responsible for anemia due to the disturbance caused to the production of Hb, and this in turn eventually causes iron deficiency anemia (Shirmohamadi et al., 2016).

#### Periodontal Pathogens and Inflammatory Cytokines Facilitate the Secretion of Ferritin

Although ferritin could not be included in this meta-analysis due to the significant heterogeneity, it plays an important role in the development of periodontitis. Ferritin, as an iron-binding protein, plays a vital role in iron storage and recycling and protects the host from infection. It consists of two subunits, the heavy chain (21 kPa) and the light chain (19 kPa) (Huang et al., 2019). It has been demonstrated that elevated ferritin levels are present in atherosclerosis, neurodegenerative disease, and even cancer (Torti and Torti, 2002). It is noteworthy that ferritin has a correlation with the development of chronic periodontal diseases. Outcomes of this study suggest that the level of serum ferritin increases in chronic periodontitis. Sufficient evidence demonstrated that not only serum but also salivary ferritin was higher in patients with chronic periodontitis, when compared to healthy controls (Guo et al., 2018). Another study showed that ferritin levels in the GCF of female patients with anemia decreased after 3 months of periodontal treatment (Enhos et al., 2009).

Abundant ferritin in the inflamed periodontal tissues in clinical and animal studies amplified the innate immune response. In addition to *P. gingivalis* lipopolysaccharide, cytokines [interleukin (IL)-1 and tumor necrosis factor alpha (TNF-α)] in the human periodontal ligament cells were also reported to facilitate the secretion of ferritin. Likewise, ferritins promote the expression of IL-6 and IL-8 through transferrin receptor-1 via extracellular signal-regulated kinase/P38 mitogen-activated protein kinase pathways that play an important role in this phase (Huang et al., 2019).

#### Periodontitis Inhibits the Proliferation and Differentiation of Erythrocytes

Periodontal pathogens, primarily Gram-negative bacteria, initiate the host's immune response and cause periodontal tissue damage (Escobar et al., 2018). In addition, the inflammatory cytokines, which are released or secreted from the affected periodontal tissue, are involved in inhibiting the proliferation and differentiation of erythrocytes (Anand et al., 2014).

With the activation of the immune system by chronic inflammation in periodontitis, a mass secretion of cytokines and bacterial lipopolysaccharide occur to promote the development of AI. These cytokines (IL-1, IL-6, IL-10, TNF-α, and interferon gamma) may enhance iron storage in macrophages by increasing the production of ferritin and damaging the erythrocytes. Apart from shortening the erythrocyte half-life, inflammatory mediators suppress the response of EPO and inhibit the differentiation of the erythroid cell, which contribute to the eventual occurrence of anemia (Weiss et al., 2019). Interestingly, inflammatory cytokines such as IL-1α, IL-6, and TNF-α are involved in the suppression of erythropoiesis (Gokhale et al., 2010). All these above phenomena imply that PD increases the risk of AI.

#### Strengths and Limitations

The main strength of this meta-analysis is the larger sample size when compared to previous studies that analyzed the association between periodontitis and anemia. In addition, secondary analysis regarding the gender, extent of periodontitis, and sample size provides a deeper understanding of the pooled result. Last but importantly, analysis of the iron metabolism biomarkers reflecting AI was conducted to evaluate the alteration of iron markers in periodontitis.

Since the studies included in this systematic review and meta-analysis are cross-sectional and qualitative information about individual anemia were absent in the included data, no strong inference could be deduced on the causality between periodontitis and AI. Furthermore, the criteria for the diagnosis of periodontitis varied among the included studies, causing the discrepancy in the extent of periodontitis in eligible subjects. Nearly half of the included studies have a small sample size and lack gender differentiation, which may have impacted the pooled result. Further research, more powered prospective observational studies, and rigorous eligible criteria with a larger sample are urgently needed.

## Conclusions

In summary, the current evidence demonstrates that periodontitis decreases Hb concentration and disturbs the balance of iron metabolism, which confirms strength of association between periodontitis and the development tendency of AI, especially for severe periodontitis. More unbiased cohort studies with larger sample sizes are still warranted to make a definitive judgment in the future.

## Author Contributions

DW and XZ designed this study. ZL, SZ, and FC retrieved the data. DL and FC analyzed the data. DW drafted the manuscript.

### Conflict of Interest

The authors declare that the research was conducted in the absence of any commercial or financial relationships that could be construed as a potential conflict of interest.
