# Supplementary material for: Decreased Hemoglobin Concentration and Iron Metabolism Disorder in Periodontitis: Systematic Review and Meta-Analysis
Source: Front Physiol. 2020 Jan 31;10:1620. doi: 10.3389/fphys.2019.01620 (PMC7005133; doi:10.3389/fphys.2019.01620)
Supplement: Supplementary file 1 [file Data_Sheet_1.pdf]

Supplementary Material

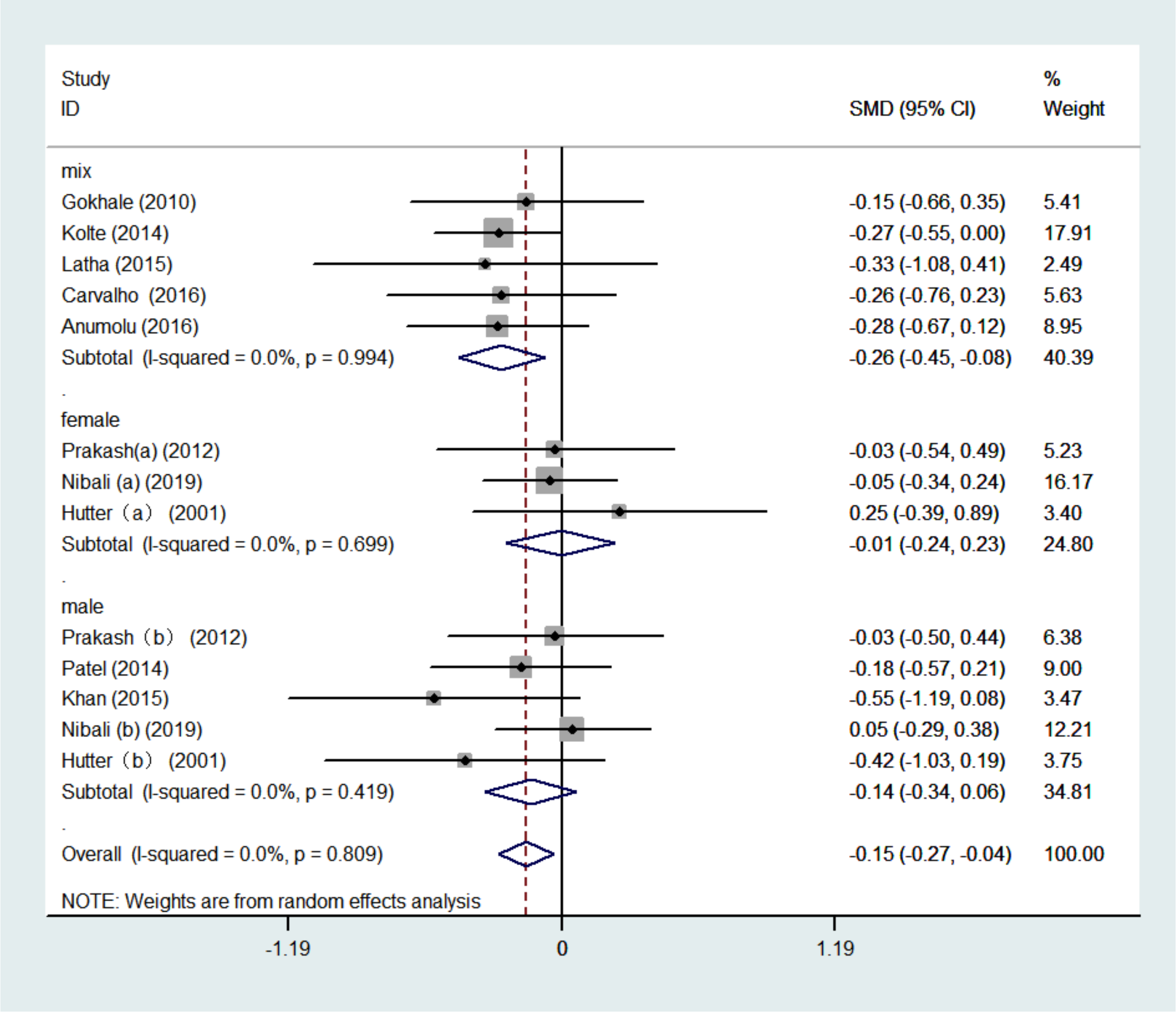

Supplementary Figure 1. Forest plot of the studies regarding the effect of periodontitis on MCH based on gender.

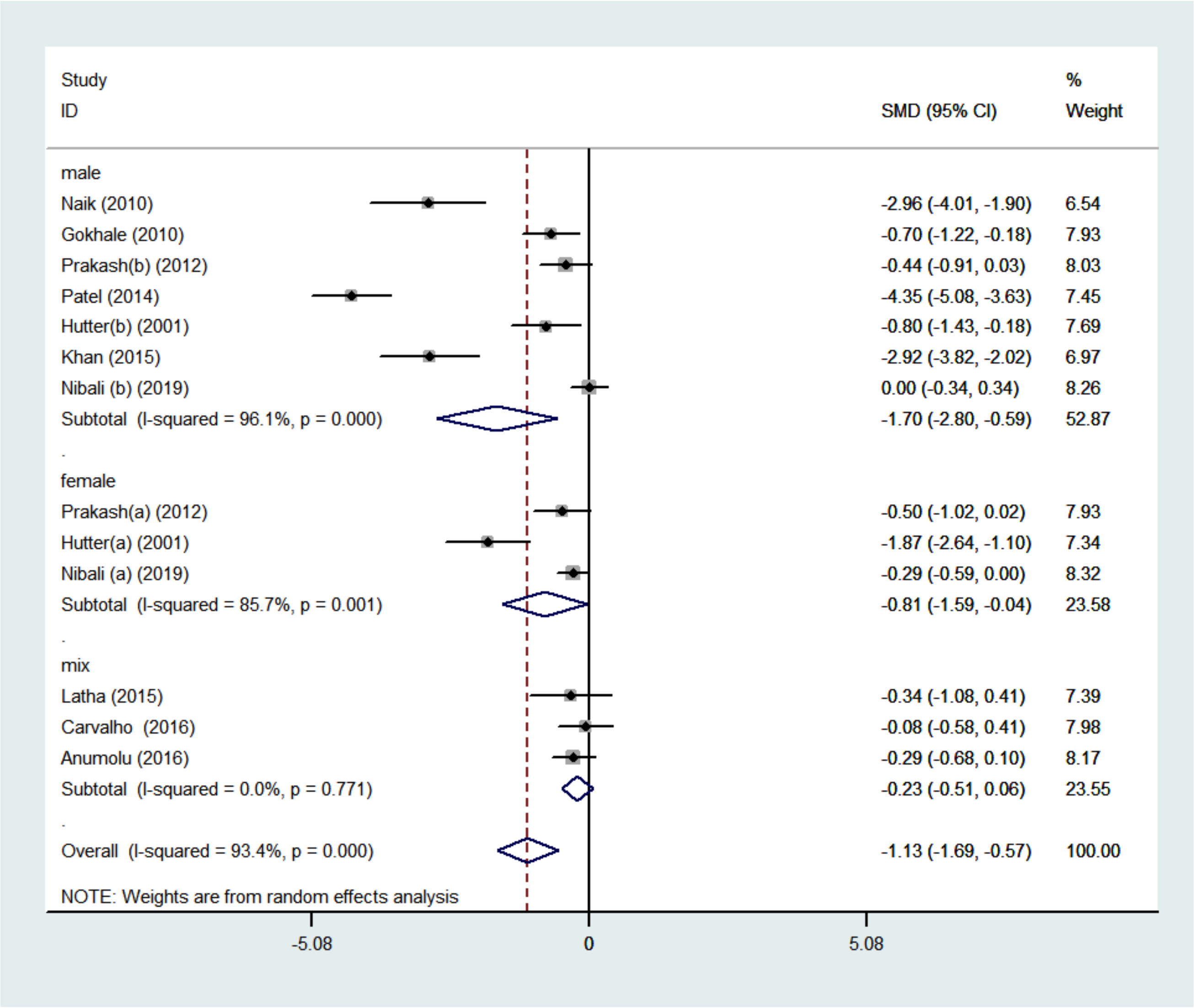

Supplementary Figure 2. Forest plot of the studies regarding the effect of periodontitis on HCT based on gender.

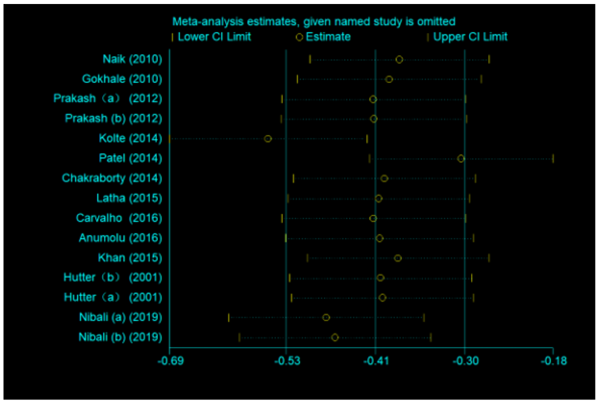

(a)

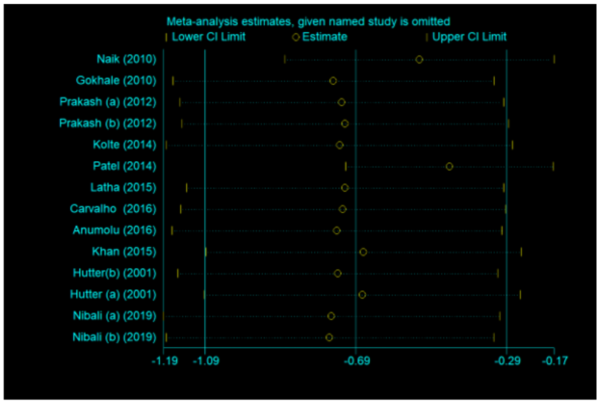

(b)

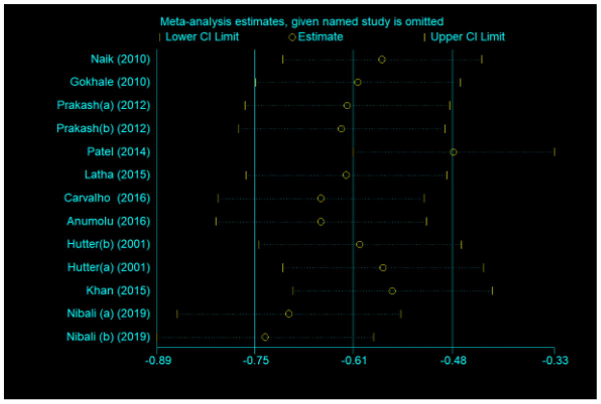

(c)

Supplementary Figure 3. Sensitivity analysis of meta-analysis.

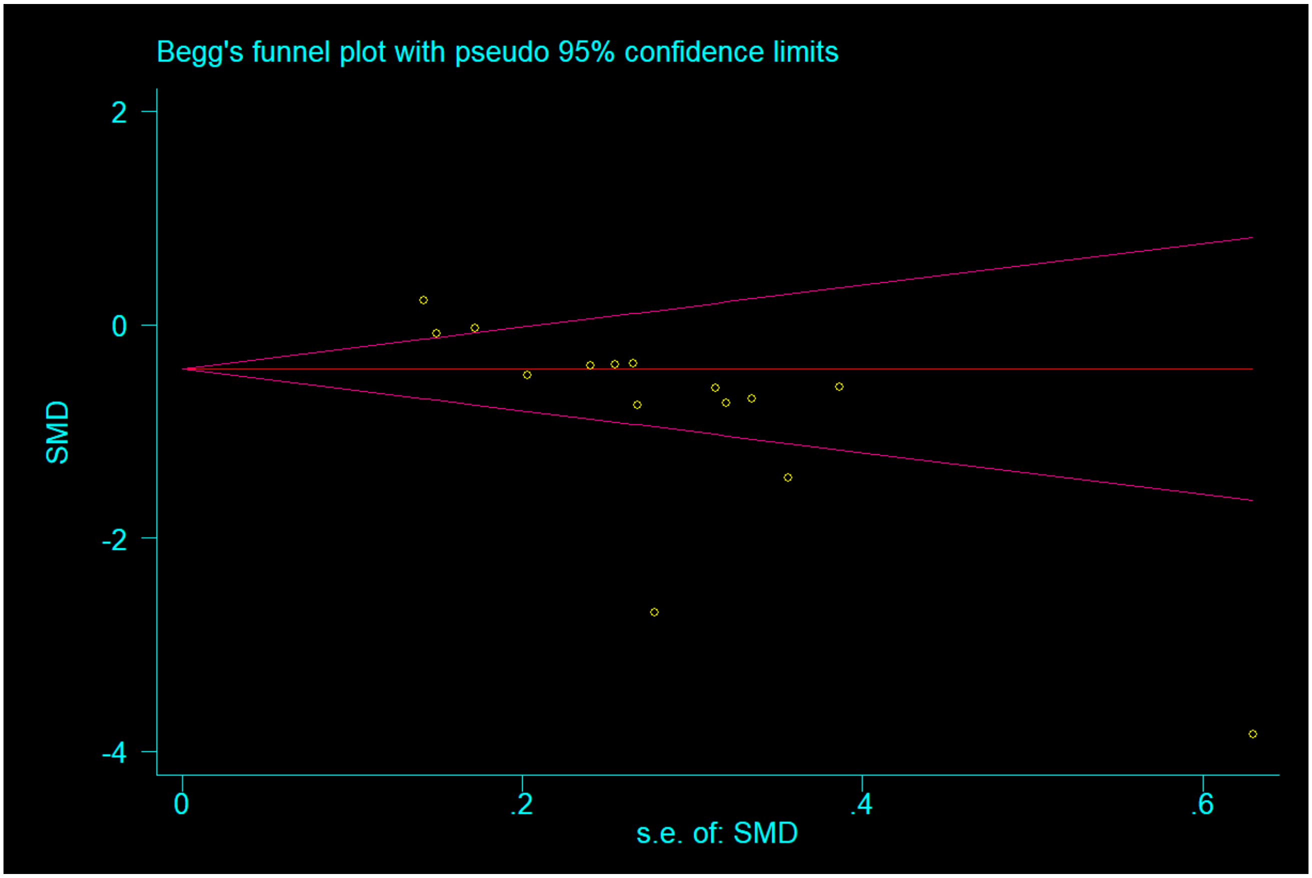

Supplementary Figure 4. Publication bias of the studies regarding the effect of periodontitis on Hb level.

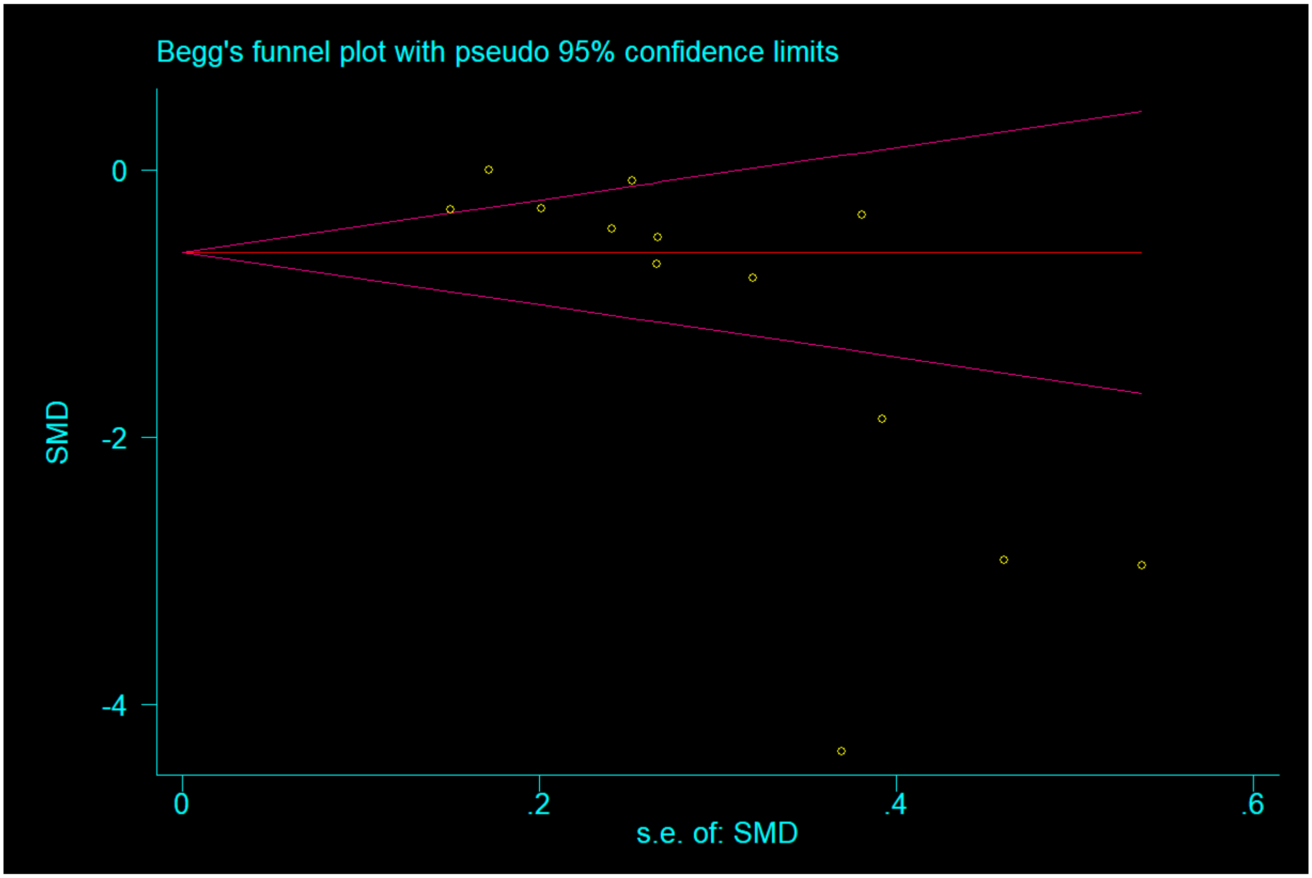

Supplementary Figure 5. Publication bias of the studies regarding the effect of periodontitis on HCT level.

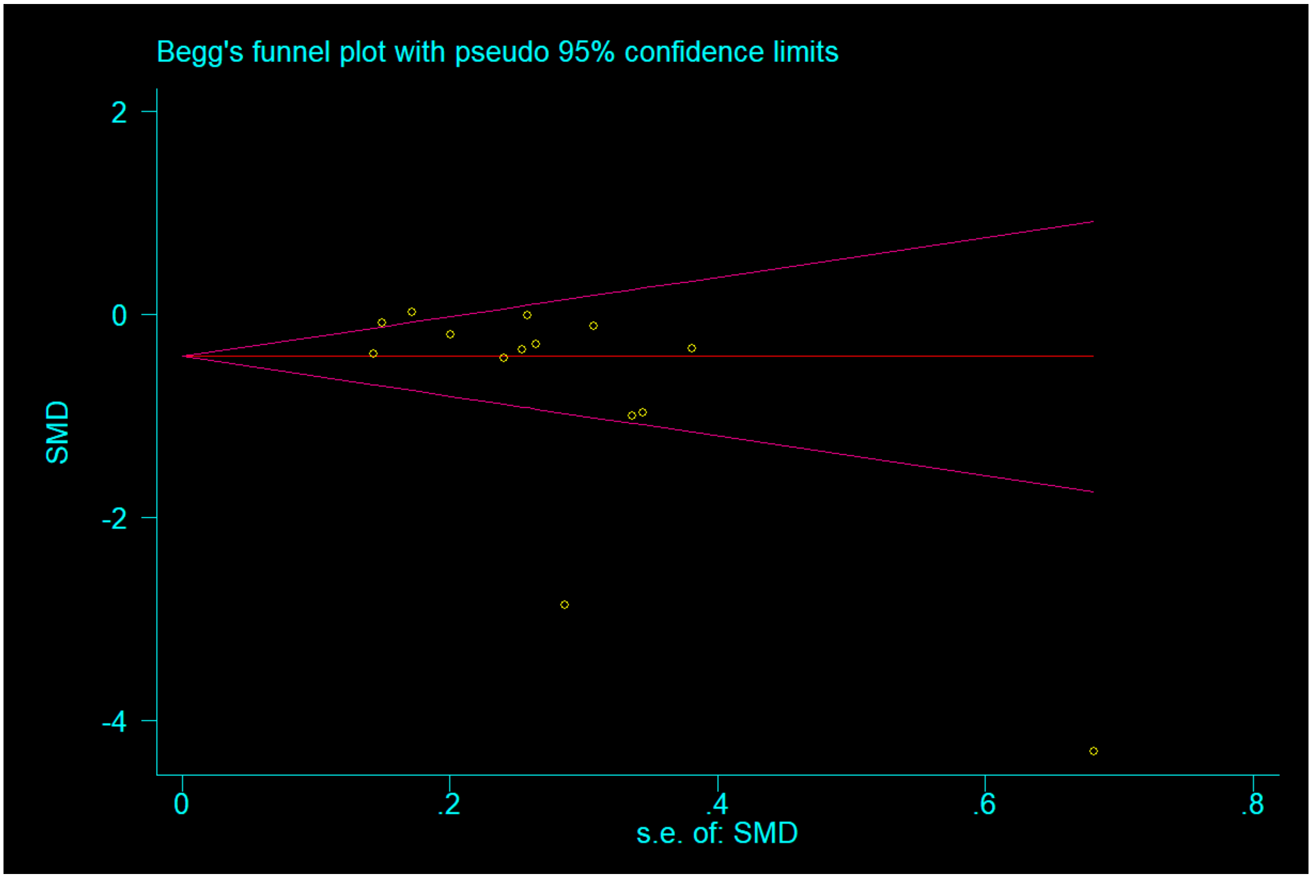

Supplementary Figure 6. Publication bias of the studies regarding the effect of periodontitis on RBC level.

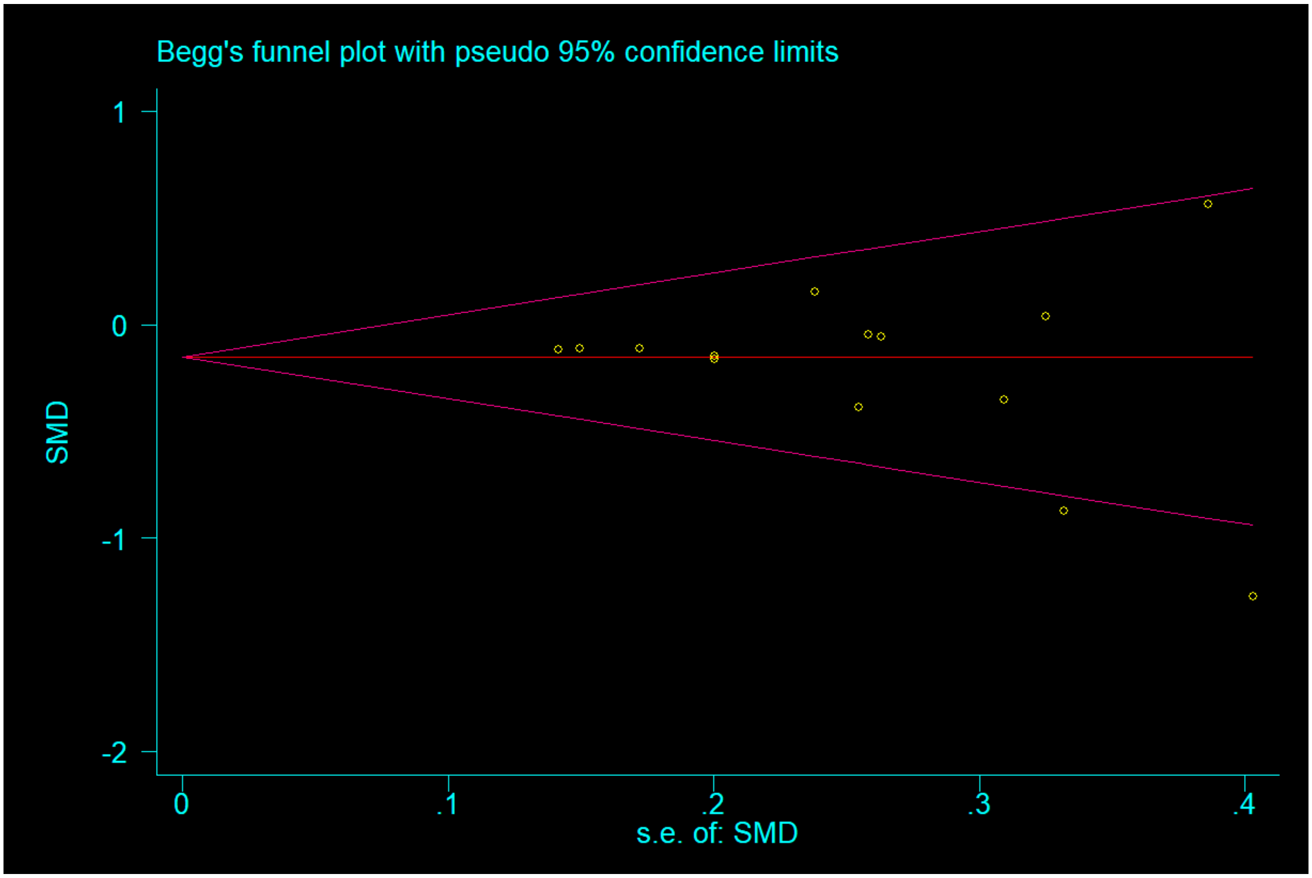

Supplementary Figure 7. Publication bias of the studies regarding the effect of periodontitis on MCV level.

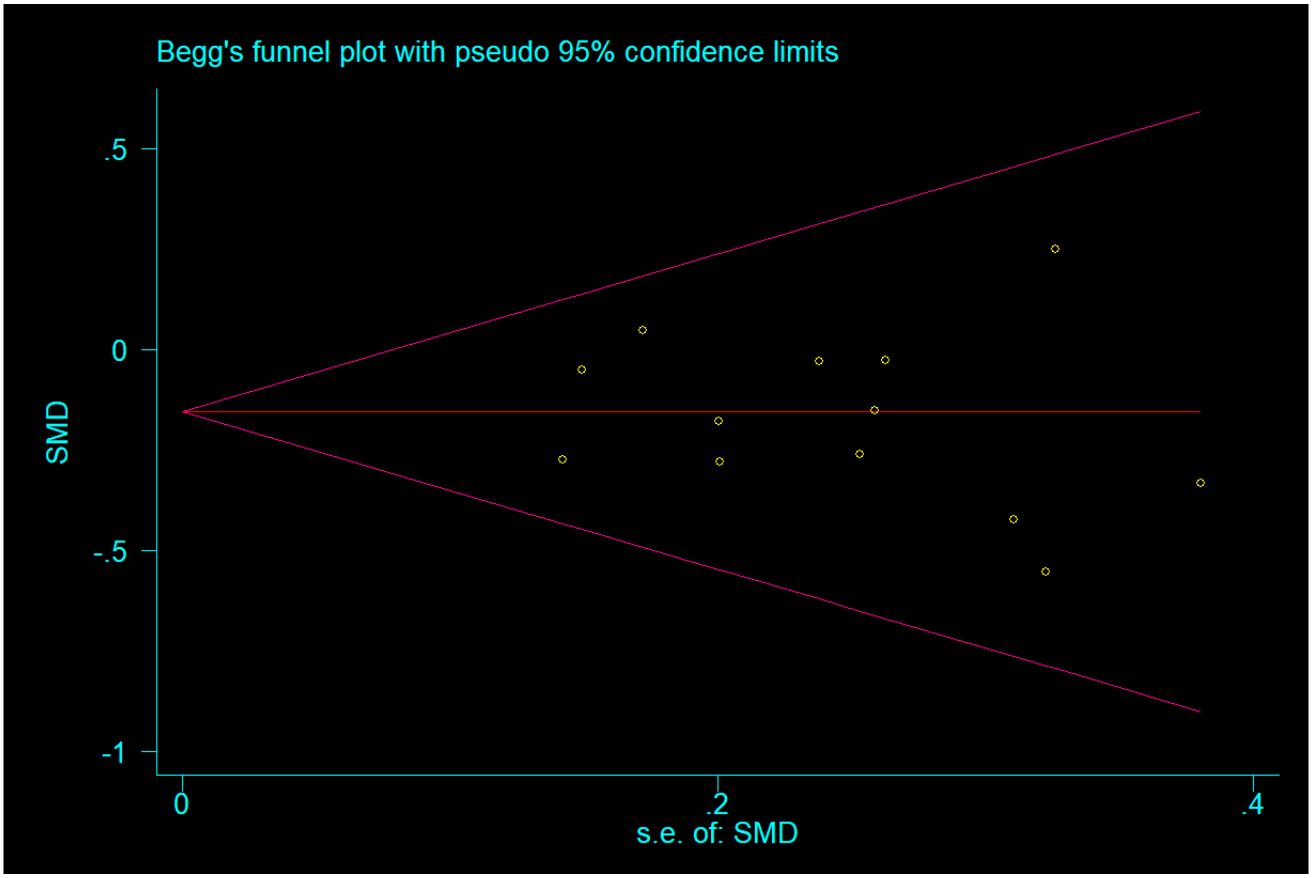

Supplementary Figure 8. Publication bias of the studies regarding the effect of periodontitis on MCH level.

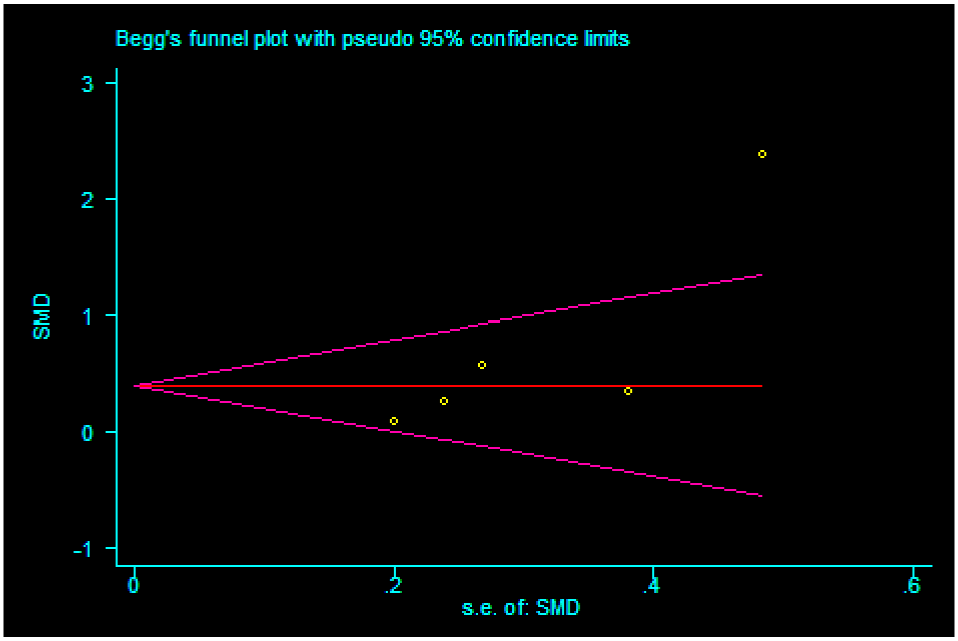

Supplementary Figure 9. Publication bias of the studies regarding the effect of periodontitis on ESR level.

Supplementary Table 1. Subgroup analysis of the association between periodontitis and anemia based on Hb level.

Subgroup	Number of subjects	SMD (95%CI)	I^2	P value
**Gender**				
Male	7	-1.14(-2.05，-0.24)	94.8%	0.01
Female	3	-0.37(-0.83，0.09)	**63.7%**	**0.11**
Mix	4	-0.33 (-0.53，-0.13)	0.00%	＜0.01
**Severity of periodontitis**				
CAL⩾5 mm	5	-0.85(-1.57, -0.13)	91.0%	0.02
BL⩾50%	3	-0.33(-0.89, 0.22)	**62.1%**	**0.24**
CAL⩾1 mm	6	-0.75(-1.45, -0.05)	93.1%	0.04
**Sample**				
＜50	5	-1.20(-2.16, -0.24)	88.1%	0.02
⩾50	9	-0.49(-0.93, -0.05)	90.9%	0.03
Total	11	-0.69(-1.09，-0.29)	89.8%	＜0.01

Supplementary Table 2. Subgroup analysis of the association between periodontitis and anemia based on RBC.

Subgroup	Number of subjects	SMD (95%CI)	I^2	P value
**Gender**				
Male	6	-0.13(-0.69, 0.00)	**63.9%**	0.05
Female	2	-0.08(-0.32, 0.16)	0.00%	0.51
Mix	3	-0.11(-0.31, -0.09)	8.50%	0.27
**Severity of periodontitis**				
CAL⩾5 mm	5	-0.43(-0.78, -0.08)	**65.7%**	**0.02**
BL⩾50%	3	-0.12(-0.45,0.22)	0.00%	0.50
CAL⩾1 mm	6	-0.06(-0.22, 0.11)	0.00%	0.51
**Sample**				
＜50	3	-0.38(-0.83, 0.08)	66.3%	0.11
⩾50	8	-0.09(-0.28, -0.01)	0.00%	0.16
Total	9	-0.16(-0.22，-0.04)	34.0%	0.03

Supplementary Table 3. Subgroup analysis of the association between periodontitis and anemia based on MCV.

Subgroup	Number of subjects	SMD (95%CI)	I^2	P value
**Gender**				
Male	7	-1.70（-2.80，-0.59）	96.1%	＜0.01
Female	3	-0.81（-1.59，-0.04）	85.7%	0.04
Mix	3	-0.23（-0.51，-0.06）	0.00%	0.12
**Severity of periodontitis**				
CAL⩾5 mm	5	-1.11(-1.95, -0.26)	93.3%	0.01
BL⩾50%	3	-1.08(-2.64, -0.42)	**69.3%**	＜0.01
CAL⩾1 mm	5	-1.17(-2.40, -0.06)	96.1%	0.06
**Sample**				
＜50	5	-1.73(-2.74, -0.73)	87.4%	＜0.01
⩾50	8	-0.79(-1.43, -0.14)	94.3%	0.04
Total	11	-1.13(-1.69，-0.57)	93.4%	＜0.01

Supplementary Table 4. Subgroup analysis of the association between periodontitis and anemia based on HCT.

Subgroup	Number of subjects	SMD (95%CI)	I^2	P value
**Gender**				
Male	6	-0.14 (-0.34, 0.06)	0.00%	0.18
Female	2	-0.01(-0.24, 0.23)	0.00%	0.97
Mix	3	-0.26(-0.45, -0.08)	0.00%	＜0.01
**Severity of periodontitis**				
CAL⩾5 mm	6	-0.10(-0.31, 0.10)	6.90%	0.32
BL⩾50%	3	-0.12(-0.48,0.23)	11.3%	0.50
CAL⩾1 mm	4	-0.20(-0.37, -0.04)	0.00%	0.02
**Sample**				
＜50	3	-0.19(-0.47, 0.09)	6.10%	0.16
⩾50	8	-0.15(-0.28, -0.01)	**0.00%**	**0.03**
Total	9	-0.15(-0.27，-0.04)	0.00%	＜0.01

Supplementary Table 5. Subgroup analysis of the association between periodontitis and anemia based on MCH.

Subgroup	Number of subjects	SMD (95%CI)	I^2	P value
**Gender**				
Male	7	-1.31(-2.14，-0.48)	93.8%	＜0.01
Female	3	-0.29(-0.62，0.05)	**36.5%**	**0.10**
Mix	5	-0.34(-0.74，0.07)	73%	0.10
**Severity of periodontitis**				
CAL≥5 mm	5	-0.93(-1.66, -0.19)	91.3%	0.01
BL≥50%	3	-0.69(-1.03, -0.35)	**0.00%**	**＜0.01**
CAL≥1 mm	7	-0.71(-1.39, -0.02)	93.3%	0.04
**Sample**				
＜50	7	-1.04(-1.61, -0.47)	80.2%	＜0.01

⩾50	8	-0.55(-1.07, -0.03)	92.7%	0.04
Total	12*	-0.76 (-1.15, -0.37)	89.9%	＜0.01
